# Susceptible trichostrongyloid species mask presence of benzimidazole-resistant *Haemonchus contortus* in cattle

**DOI:** 10.1186/s13071-021-04593-w

**Published:** 2021-02-08

**Authors:** Khalid M. Mohammedsalih, Jürgen Krücken, Ahmed Bashar, Fathel-Rahman Juma, Abdalhakaim A. H. Abdalmalaik, Amna Khalafalla, Adam Abakar, Gerald Coles, Georg von Samson-Himmelstjerna

**Affiliations:** 1 Faculty of Veterinary Science, University of Nyala, P.O. Box 155, Nyala, Sudan; 2 Institute for Parasitology and Tropical Veterinary Medicine, Freie Universität Berlin, Robert-von-Ostertag-Street 7-13, 14163 Berlin, Germany; 3 Faculty of Veterinary Medicine, University of Khartoum, P.O. Box 32, Khartoum North, Sudan; 4 Faculty of Medical Laboratory Sciences, University of Gezira, P.O. Box 20, Wadmedani, Sudan; 5 Ubley Biologics, Ubley, P.O. Box 170, Bristol, BS40 6JA UK

**Keywords:** β-tubulin, Anthelmintic resistance, Gastro-intestinal nematodes, *Haemonchus placei*

## Abstract

**Background:**

Benzimidazole (BZ) anthelmintics are widely used to control infections with parasitic nematodes, but BZ resistance is an emerging threat among several nematode species infecting humans and animals. In Sudan, BZ-resistant *Haemonchus contortus* populations were recently reported in goats in South Darfur State. The objective of this study was to collect data regarding the situation of BZ resistance in cattle parasitic nematodes in South Darfur using phenotypic and molecular approaches, besides providing some epidemiological data on nematodes in cattle.

**Methods:**

The faecal egg count reduction test and the egg hatch test (EHT) were used to evaluate benzimidazole efficacy in cattle nematodes in five South Darfur study areas: Beleil, Kass, Nyala, Rehed Al-Birdi and Tulus. Genomic DNA was extracted from pools of third-stage larvae (L3) (*n *= 40) during trials, before and after treatment, and pools of adult male *Haemonchus* spp. (*n *= 18) from abattoirs. The polymorphisms F167Y, E198A and F200Y in isotype 1 β-tubulin genes of *H. contortus* and *H. placei* were analysed using Sanger and pyrosequencing.

**Results:**

Prevalence of gastro-intestinal helminths in cattle was 71% (313/443). Reduced albendazole faecal egg count reduction efficacy was detected in three study areas: Nyala (93.7%), Rehed Al-Birdi (89.7%) and Tulus (88.2%). In the EHT, EC_50_ values of these study areas ranged between 0.032 and 0.037 µg/ml thiabendazole. Genus-specific PCRs detected the genera *Haemonchus*, *Trichostrongylus* and *Cooperia* in L3 samples collected after albendazole treatment. Sanger sequencing followed by pyrosequencing assays did not detect elevated frequencies of known BZ resistance-associated alleles in codon F167Y, E198A and F200Y in isotype 1 β-tubulin gene of *H. placei* (≤ 11.38%). However, polymorphisms were detected in *H. contortus* and in samples with mixed infections with *H. contortus* and *H. placei* at codon 198, including E198L (16/58), E198V (2/58) and potentially E198Stop (1/58). All pooled L3 samples post-albendazole treatment (*n *= 13) were identified as *H. contortus* with an E198L substitution at codon 198.

**Conclusions:**

To the knowledge of the authors, this is the first report of reduced albendazole efficacy in cattle in Sudan and is the first study describing an E198L substitution in phenotypically BZ-resistant nematodes collected from cattle.
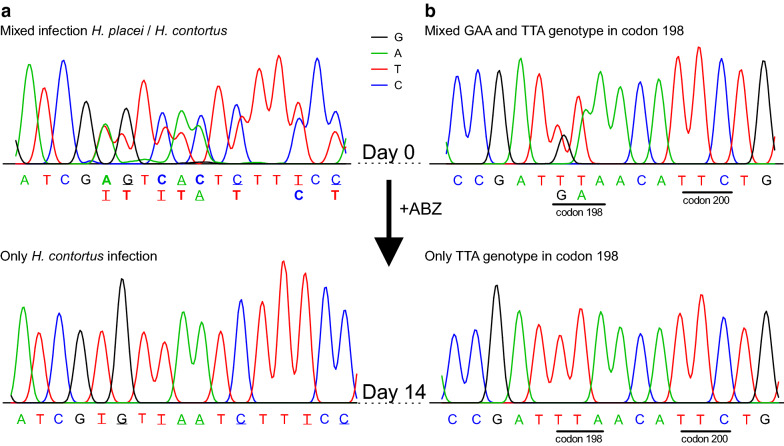

## Background

Infections with parasitic nematodes have a significant impact on global health and socioeconomic development. In tropical countries, including Sudan, parasitic nematodes are among the most widespread pathogens of humans and animals [1, 2]. *Haemonchus contortus* and *Haemonchus placei* are ranked among the top pathogenic species of gastro-intestinal nematodes (GINs) of ruminants because of their haematophagous feeding behaviour resulting in anaemia, hypoproteinaemia, loss of animal live-weight and death in heavy infections, particularly in young animals [3]. *Haemonchus placei* is generally recognized as a cattle parasite but infections with both species, *H. contortus* and *H. placei*, occasionally do occur in younger cattle and cause similar disease as in sheep and goats [4].

Globally, control of parasitic nematode infections in humans and animals relies on the efficacy of a limited range of broad-spectrum anthelmintics such as benzimidazoles (BZs). Since introduced in the market over 50 years ago [5], BZs (e.g. albendazole) have frequently been used in treatment and control programmes against GIN infections all over the world [2, 4]. The widespread use of this drug class is due to a broad-spectrum nematocidal efficacy and a safety margin generally preventing side effects due to overdosing. As a consequence of selection due to the intensive use of BZs over several decades, parasitic nematode populations have evolved that display anthelmintic resistance. Currently, BZ resistance is a significant problem in veterinary medicine [6]. The presence of nematode populations displaying anthelmintic resistance in the field increases the economic losses due to persistent sub-clinical parasitism, the additional cost of re-diagnosis of infected animals and the change of anthelmintic classes [7]. Recently, 38 million euros annually was the estimated cost associated with anthelmintic resistance in parasitic nematodes assessed for 18 European countries [8]. In this context it is important to improve the current knowledge of anthelmintic resistance mechanisms to facilitate better diagnostic tools for field surveys and update drug treatment strategies [6, 9].

The molecular mechanisms of BZ resistance have been intensively studied in numerous nematodes including *H. contortus* and *H. placei* from different geographical regions [6, 10, 11]. Benzimidazole resistance has long been associated with the occurrence of three-point mutations in the isotype 1 β-tubulin gene, including a substitution of phenylalanine by tyrosine at codon positions F167Y [12] and F200Y [13], and a change at codon position 198 from glutamate to alanine (E198A) [14]. In recent years, through the improvement of sequencing technologies, additional new alleles at codon 198 of isotype 1 β-tubulin gene, also correlated with BZ resistance, in particular E198L and E198V, have been identified [15–19].

In Sudan, BZ-resistant *H. contortus* populations were identified in goats in South Darfur at the phenotypic [20] and molecular level by the identification of five different substitutions at codon position 198 of the isotype 1 β-tubulin gene, i.e. E198L, E198V, E198K and potentially E198I or E198Stop. The E198L substitution was detected in 100% of the *H. contortus* samples that survived different treatment strategies with albendazole [19].

Cattle are central to the economy and the society of Sudan including South Darfur State where the cattle population is estimated to be around 4 million [21], but there is little information on the control of GINs in cattle. Therefore, this study was undertaken to collect data regarding the situation of BZ resistance in cattle parasitic nematodes in South Darfur at the phenotypic level to specifically understand the molecular mechanisms of resistance with particular focus on the most pathogenic species, *H. contortus* and *H. placei*, and to provide some epidemiological data on nematodes in cattle.

## Materials and methods

### Study location and design

The study was conducted in South Darfur State (11.30°N, 24.40°E), southwestern Sudan, in five different study areas. The selected areas were Beleil (12.02°N 24.99°E), Kass (12.50°N 24.28°E), Nyala (12.05°N 24.88°E), Rehed Al-Birdi (11.30°N 23.88°E) and Tulus (11.00°N 2.00°E). South Darfur is in a savannah zone with a very long dry season and only a single rainy season from June to November, the highest precipitation occurring between July and September (range 377–546 mm/month) with mean minimum and maximum temperatures of 24.7–37.6 °C and mean minimum and maximum relative humidity of 28.3–56.7% [22]. Open grazing is the main husbandry system. Baggara cattle are the predominant breed [21].

Albendazole efficacy and BZ resistance in cattle were evaluated using both phenotypic [faecal egg count reduction test (FECRT) and egg hatch test (EHT)] and molecular techniques. The field trials including collection of all samples in different study areas were performed during the rainy seasons (June–November) in the years 2016–2018.

The size of cattle farms in the selected areas was always very small (mostly only up to seven animals per owner), and cattle belonging to different owners frequently shared pastures. Therefore, albendazole efficacy was studied on a regional and not a farm level.

### Albendazole treatment in cattle naturally infected with gastro-intestinal strongyles

In the five South Darfur study areas, 88 farms were selected randomly, each farm had 2–14 head of cattle, and the farms were distributed as follows: Beleil (*n *= 13), Kass (*n *= 14), Nyala (*n *= 23), Rehed Al-Birdi (*n *= 16) and Tulus (*n *= 22). A total of 443 cattle of both sexes and different age groups that had not been treated with any anthelmintic for at least 1 month were selected for collection of faecal samples to be examined for the presence of infections with GINs.

Cattle (*n *= 123) were selected that were positive for infection with strongyle nematodes and had ≥ 150 eggs per gram (epg) faeces [23]. The selected cattle were from 55 out of 88 farms [Beleil (*n *= 8), Kass (*n *= 9), Nyala (*n *= 12), Rehed Al-Birdi (*n *= 12) and Tulus (*n *= 14)], and each selected farm had 1–7 heads. Both sexes and different age groups (< 1 year: young; > 1 years: adult, based on dentition [24]) were represented in the study population and grouped into control (*n *= 41) and treatment (*n *= 82) groups. Animals were assigned arbitrarily to each experimental group at the level of the five regions mentioned above to achieve a ratio of control/treatment animals of 1:2 if ≥ 15 animals were available and of 1:1 if the number of animals was < 15. Furthermore, if the number of animals in any farm was higher than one, this farm was represented in the two experimental groups. Before albendazole treatment, the body weight of each animal was determined by the measurement of heart girth and body length [25] and was calculated using the formula:

$${\text{Heart}}\;{\text{girth}} \times {\text{heart}}\;{\text{girth }} \times {\text{ body}}\;{\text{length}}/600 = {\text{animal}}\;{\text{weight}}\;{\text{in}}\;{\text{kilograms}}{.}$$

Albendazole commercial brand [Albex^®^ 10% w/v oral suspension (batch No. H30275; Chanelle, Hungerford, UK)] was used orally at 7.5 mg/kg body weight. The 82 treated animals were distributed among the regions as follows: Beleil (11), Kass (11), Nyala (20), Rehed Al-Birdi (20) and Tulus (20). Faecal samples were collected before treatment (day 0) and then on day 8 and 14 after drug administration. The tested animals stayed in their herds throughout the experiment and after the testing was finished.

### Faecal sample analyses

#### Faecal egg counts

Individual rectal faecal samples were collected in plastic bags, labelled and stored at 4 °C for a maximum of 24 h before egg counting. Samples from trials in Nyala and Beleil were analysed at the Laboratory of Parasitology, Faculty of Veterinary Science, University of Nyala, Sudan, while those from Kass, Rehed Al-Birdi and Tulus were analysed directly in the field. For egg counting, the Fill-FLOTAC and the Mini-FLOTAC devices, using saturated sodium chloride solution as flotation medium, were used with a theoretical sensitivity of 5 epg [26]. The eggs of different helminths infecting ruminants were identified as described previously [27].

#### Faecal cultures

For faecal cultures on days 0 and 14, samples were pooled on the farm level. Faeces were mixed with wood shaving and incubated in labelled plastic jars at 22–27 °C with daily moistening using distilled water for 10 days [27]. Then, L3 were purified using the Baermann funnel method. Microscopical differentiation used 100 L3 that were assigned to *Haemonchus* spp., *Trichstrongylus* spp. and *Oesophagostomum/Chabertia* spp. according to published differentiation keys [27, 28].

#### Egg hatch test (EHT)

The EHT was conducted as recommended by the guidelines of the World Association for the Advancement of Veterinary Parasitology (WAAVP) [23, 29]. On day 0, fresh faecal samples were pooled at the region level; nematode eggs were extracted [30] and used within 4 h. In Tulus, day 14 faecal samples from albendazole-treated cattle were used in addition to day 0 samples.

### Statistical analyses

Data from the survey for the presence of gastro-intestinal helminth infections in cattle in the five selected South Darfur study areas were used to estimate the prevalence with the infection with strongyle nematodes in the tested areas using R software version 3.5.3 and the graphical user interface RStudio version 1.2.5019. The glm.nb function from the MASS package was used to perform negative binomial regressions for egg count data. As explanatory variables, the sex, the age group (young animals vs. adult) as well as an interaction between sex and age group were considered. Initially, a full model with all potential explanatory variables mentioned above was calculated. Then, variables were stepwise eliminated using the drop1 function aiming to improve (minimise) the Akaike information criterion (AIC). Risk ratios with 95% confidence intervals (CIs) were calculated by applying the confint function on the model coefficients. The RsqGLM function from the modEvA package was used to determine Nagelkerke pseudo R2 values.

The efficacy of albendazole was determined using the FECRT by comparing faecal egg count data from days 8 or 14 between control and treatment groups. For paired and unpaired analyses, FECs on these days were either compared with data from the same animal on day 0 or with data from the control group on the same days post-treatment. To calculate paired and unpaired estimates of the FECR with 95% CIs, the R package eggCounts version 1.1–1 [31] was used with the zero-inflation option.

Resistance to BZs was considered present when the FECR and its upper 95% CI were < 95% and the lower 95% CI was < 90%. Parasites were considered to be susceptible when the FECR was > 95% and its lower 95% CI was > 90%. Otherwise, the FECRT was considered to be inconclusive [29, 32].

For the EHT, EC_50_ values were calculated using four parameter logistic regression analysis in GraphPad Prism version 6.01. As the EC_50_ cut-off value for BZ resistance in cattle has not been established for nematode eggs, the value for sheep nematodes was assumed to apply (> 0.1 μg thiabendazole/ml) [23, 29].

The statistical relationship between the variables FECRT and EHT was calculated using the non-parametric Spearman correlation test in GraphPad Prism version 6.01. All sets of data obtained on FECRT and EHT for BZ resistance were analysed to observe correlation among each other through linear regression analysis. For the FECRT, the day 14 unpaired data were used.

### Parasite material for molecular analyses

#### Third-stage larvae

Forty samples, each containing at least ~ 1000 nematode L3, were collected, each sample consisting of L3 pooled from at least two animals. The samples were collected from all cattle (*n *= 123) included in the trials, which were derived from 55 farms in the five study areas. The preparation of pooled samples depended on the number of animals in the control or treatment group on each farm: if only a single animal was present, pooled samples were prepared from animals derived from two or more farms that shared the same pasture for grazing. If two or more animals from the same group were present on a farm, L3 were pooled from this farm. Cattle treated with albendazole that showed FECs of zero on day 14 were not included in the preparation of pooled L3 samples after albendazole treatment. Therefore, the 40 samples were composed of 27 pooled L3 samples from day 0 and 13 from day 14 after albendazole treatment. Nematode L3 were preserved in 70% ethanol.

#### *Adult male Haemonchus *spp.

The abattoirs in Kass, Nyala, Rehed Al-Birdi and Tulus were visited twice 7 days apart, and all cattle abomasa were inspected for the presence of *Haemonchus* spp. Cattle abomasa [*n *= 18: Kass (0), Nyala (3), Rehed Al-Birdi (4) and Tulus (11)] from animals with unknown history of anthelmintic treatment were sampled and all adult male *Haemonchus* spp. (16–100 from each abomasum) were isolated for each individual cattle. Worms were preserved in 80% ethanol.

### Genomic DNA isolation

Ethanol was removed from pooled L3 or adult samples by washing with distilled water five times. NucleoSpin^®^ Soil DNA extraction kit and NucleoSpin^®^ DNA Tissue extraction kit (Macherey-Nagel, Düren, Germany) were used for extraction of genomic DNA from L3 and adult samples, respectively. The manufacturer’s protocol for extracting DNA was followed. Details are available in Mohammedsalih et al. [19].

### Genus-specific PCRs

This technique, targeting the internal transcriber spacer 2 regions, was used to detect *Haemonchus* spp., *Trichostrongylus* spp., *Cooperia* spp., *Teladorsagia* spp. and *Ostertagia* spp. in pooled L3 samples from day 0 (*n *= 27) and day 14 (*n *= 13) after treatment with albendazole. PCRs were performed as described previously [19, 33].

### Sanger sequencing of *Haemonchus* spp. isotype 1 β-tubulin gene fragments

PCR followed by Sanger sequencing was used as a qualitative tool to inspect for the presence of mutations in either of the three codon positions 167, 198 and 200 in the isotype 1 β-tubulin gene of *Haemonchus* spp. using pooled L3 samples (*n *= 40) and pooled adult male *Haemonchus* spp. samples from abattoirs (*n *= 18). PCR was performed using previously described primers and reaction conditions [34, 35]. The reactions were recently detailed in Mohammedsalih et al. [19]. PCR products were purified and sent for Sanger sequencing (LGC Genomics, Berlin, Germany). Sequencing results were submitted to BLASTn searches and manually analysed in BioEdit software version 7.2.6 [36] to identify the species, i.e. *H. contortus*, *H. placei* or mixed infections with *H. contortus* and *H. placei* [37]. The same chromatograms were further inspected for the presence of exchanges at codons 167, 198 and 200 as detailed recently [19, 34].

### Pyrosequencing assays

In goats in Sudan [19], pyrosequencing assays failed to quantify allele frequencies of the new codon 198 variants in the isotype 1 β-tubulin gene of *H. contortus*. The assays were used to confirm results obtained by Sanger sequencing by measuring allele frequencies at codons 167 (TTC/TAC), 198 (GAA/GCA) and 200 (TTC/TAC) in the isotype 1 β-tubulin gene of *H. placei*. Eight DNA samples of pooled adult male *Haemonchus* spp. (16–100 worms/pool) [Nyala (*n *= 2), Rehed Al-Birdi (*n *= 3) and Tulus (*n *= 3)] were analysed. The assays followed a previously described protocol [34] as modified by Ademola et al. [35] and detailed in Mohammedsalih et al. [19].

## Results

### Prevalence and risk factors for gastro-intestinal helminth infections

In the five South Darfur study areas the prevalence of gastro-intestinal helminth in cattle was 71% (313/443) on the level of individual animals (Table 1). The microscopic examination of eggs identified two groups of helminths, i.e. strongyle nematodes and *Strongyloides* spp. Prevalence of strongyle nematodes was by far the highest (71%). *Strongyloides* spp. was detected only in mixed infections with strongyles, in 2% of the animals. Morphologically, the faecal cultures identified three groups of strongyle larvae, i.e. *Haemonchus* spp., *Trichostrongylus* spp. and *Oesophagostomum/Chabertia* spp., but by far the most frequent parasite genus was *Haemonchus* spp. (86%) (Table 1).

**Table 1:** Prevalence, mean egg counts and results of larval differentiation for cattle naturally infected with gastrointestinal helminth from five different study areas in South Darfur State, Sudan, during the rainy season

	All animals	Sex		Age		Study area					
		Male	Female	Young	Adult	Beleil	Kass	Nyala	Rehed Al-Birdi	Tulus	
Prevalence of the infection											
No. of the tested cattle	443	125	318	234	209	96	63	130	62	92	
No. (%) of the infected cattle	314 (71)	96 (77)	218 (69)	201 (86)	113 (54)	77 (80)	31 (49)	103 (79)	48 (77)	55 (60)	
No. (%) of strongyles	314 (71)	96 (77)	218 (69)	201 (86)	113 (54)	77 (80)	31 (49)	103 (79)	48 (77)	55 (60)	
No. (%) of cattle shedding both strongyles + *Strongyloides* spp. eggs	8 (2)	5 (4)	3 (1)	7 (3)	1 (0.5)	0 (0)	0 (0)	0 (0)	3 (5)	5 (5)	
Cattle shedding ≥ 150 strongyle epg	160 (36)	56 (45)	104 (33)	133 (57)	27 (13)	28 (29)	18 (29)	44 (34)	37 (60)	33 (36)	
Egg count/gram of positive faeces (range)											
Strongyles	338 (10–4320)	551 (20–4320)	244 (10–2860)	458 (20–4320)	123 (10–3200)	147 (20–820)	167 (10–500)	223 (20–1340)	836 (20–4320)	480 (10–3200)	
Relative strongyles species composition (%) for coprocultures^a,^ ^b^											
* Haemonchus* spp.	86					90	77	80	90	92	
* Trichostrongylus* spp.	10					10	15	15	5	5	
* Oesophagostomum*/*Chabertia* spp.	4					0	8	5	5	3	

^a^Samples pooled on a regional level. In each study area and on the day of sample collection, faecal samples pooled and cultured only from cattle shedding strongyle eggs. Third-stage larvae were harvested, strongyle larvae differentiated microscopically and then the mean of different days calculated

^b^The number of pools were two up to four from each study area

A negative binomial regression model was calculated to determine potential effects of risk factors on egg counts. This model showed that the epgs were significantly higher in the Rehed Al-Birdi and Tulus regions than in Kass (Table 2). Young animals (i.e. < 1 year) shed significantly more strongyle eggs than adults (risk ratio 5.176, *P* < 0.0001). The variable sex showed that males shed significantly more strongyle eggs than females (risk ratio 5.176, *P *< 0.05). There was also a significant interaction between the variables sex and age showing that the effect of sex was less pronounced in young animals (Table 2).

**Table 2:** Final negative binomial regression model to identify variables with influence on faecal egg counts with cattle in five different study areas in South Darfur State, Sudan, during the rainy season

Term	Estimate	Standard error	Statistic	*P* value	Risk ratio	95% Confidence interval
Intercept	3.346	0.255	13.142	< 0.0001	28.388	17.591–49.192
Study area: < Ref. Kass						
Beleil	0.522	0.320	1.631	0.1030	1.686	0.886–3.127
Nyala	0.621	0.304	2.043	0.0411	1.860	1.011–3.296
Rehed Al-Birdi	1.430	0.376	3.799	0.0002	4.177	1.999–8.756
Tulus	0.786	0.322	2.440	0.0147	2.195	1.110–4.275
Age: Ref.: adult						
Young	1.743	0.232	7.524	< 0.0001	5.716	3.640–9.097
Sex: Ref. female						
Male	1.634	0.522	3.133	0.0017	5.127	1.997–17.360
Age × sex: Ref: adult; female						
Young: male	− 1.623	0.577	− 2.810	0.0050	0.197	0.055–0.564

Nagelkerke R^2^ = 0.233

### Albendazole efficacy based on egg count data and egg hatch test

Results of the FECRT and the EC_50_ values in the EHT with 95% CIs are presented in Table 3 while Additional file 1 Table S1 shows mean egg count data with 95% CIs. Reduced albendazole efficacy was observed in three out of the five South Darfur study areas. In Tulus, albendazole was considered not effective on days 8 and 14 post-treatment when the paired data from treated cattle were analysed before and after treatment but inconclusive for the unpaired analysis. In Rehed Al-Birdi, the FECR was at least 92%; however, the 95% CIs were inconclusive for all types of analyses. The FECRT for Nyala was inconclusive on day 8 and 14 with unpaired statistics but suggested full efficacy for the paired data analysis. In Beleil and Kass, albendazole was effective (> 99%) for all types of data analyses (Table 3).

**Table 3:** Faecal egg count reduction (FECR) (and 95% confidence intervals) and the EC_50_ (and 95% confidence intervals) in the egg hatch test with cattle naturally infected with strongyle nematodes before and after oral administration of albendazole at a dose of 7.5 mg/kg body weight to the treated groups at five different study areas in South Darfur State, Sudan

Study area	No. of animals in each trial	Day 8		Day 14		EC_50_ (µg/ml thiabendazole)
		FECR (%) unpaired^a^	FECR (%) paired^a^	FECR (%) unpaired^a^	FECR (%) paired^a^	
Beleil	Control: *n* = 6	99.9 (98.7–100)	99.9 (99.5–100)	99.9 (98.8–100)	99.9 (99.5–100)	0.011^b^ (0.009–0.014)
	Treated: *n* = 11					
Kass	Control: *n* = 5	99.7 (95.8–100)	99.7 (98.9–100)	99.5 (84.1–100)	99.5 (98.3–99.9)	0.012^b^ (0.005–0.028)
	Treated: *n* = 11					
Nyala	Control: *n* = 10	95.2 (88.2–98.7)	96 (93.4–98.2)	93.7 (84.9–97.6)	94.6 (92.6–96.8)	0.032^b^ (0.023–0.046)
	Treated: *n* = 20					
Rehed Al-Birdi	Control: *n* = 10	91.3 (46.4–98.5)	92.9 (91.8–94)	89.7 (46.1–97.8)	92 (90.9–93)	0.034^b^ (0.017–0.065)
	Treated: *n* = 20					
Tulus	Control: *n* = 10	90.7 (49.8–98.8)	90.9 (89.6–92.3)	88.2 (54.0–98.4)	88.5 (87.2–90.1)	0.037^b^ (0.025–0.057) 0.113^c^ (0.036–0.356)
	Treated: *n* = 20					

^a^FECRs were calculated either by comparing data post-treatment between treatment and control group (unpaired) or between data before and after treatment (paired)

^b^Samples collected on day 0

^c^Samples collected from albendazole treated group on day 14

The EC_50_ values for thiabendazole in the EHT in the areas with reduced albendazole efficacy (Nyala, Rehed Al-Birdi and Tulus) were in a range of 0.032–0.037 µg/ml thiabendazole with nematode eggs from day 0 faecal samples. In Tulus, the EC_50_ value using nematode eggs that survived albendazole treatment of day 14 was 0.113 µg/ml thiabendazole (Table 3).

**Fig. 1:**
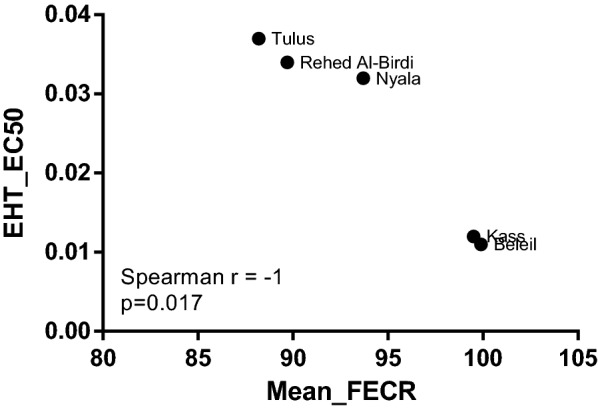
Correlation between the faecal egg count reduction test (FECRT) and the egg hatch test (EHT) for benzimidazole resistance in parasitic nematodes of cattle in five different South Darfur, Sudan, study areas. The FECRT data used were of day 14 of unpaired statistic, and the EHT performed using pooled faecal samples of day 0

Both phenotypic tests, FECRT and EHT, showed very high correlation (Spearman *ρ *= − 1, *p* = 0.017) (Fig. 1).

### Genus-specific PCRs

Samples with pooled L3 were collected pre (*n *= 27 pools of L3) and post (*n *= 13) treatment with albendazole. The parasites tested for were *Haemonchus* spp., *Trichostrongylus* spp., *Cooperia* spp., *Teladorsagia* spp. and *Ostertagia* spp. (Table 4). *Teladorsagia* spp. and *Ostertagia* spp. were not detected while *Haemonchus* spp. were present in all pre-treatment samples. *Trichostrongylus* spp. and *Cooperia* spp. were detected in 26 out of 27 pre-treatment samples. Post-treatment samples also contained all three genera, but with different frequencies than before treatment. *Haemonchus* spp. were present in all samples. *Cooperia* spp. and *Trichostrongylus* spp. were identified in 7 and 8 out of 13 samples, respectively.

**Table 4:** Detection of trichostrongyloid genera in third-stage larvae obtained from faecal samples pooled on farm level at five different study areas in South Darfur State, Sudan, before and after treatment of cattle with albendazole, using genus-specific PCR

Study area	Day of sample collection	No.^a^	No. of positive pools		
			*Haemonchus*	*Trichostrongylus*	*Cooperia*
Beleil	0	1	1	1	1
	14	n.a.	–	–	–
Kass	0	5	5	5	5
	14	1	1	1	1
Nyala	0	7	7	7	7
	14	2	2	2	2
Rehed Al-Birdi	0	9	9	9	9
	14	4	4	3	2
Tulus	0	5	5	4	4
	14	6	6	2	2
Total of pre-treatment samples (and %)		27	27 (100)	26 (96)	26 (96)
Total of post-treatment samples (and %)		13	13 (100)	8 (62)	7 (54)
Total of pre- and post-treatment samples (and %)		40	40 (100)	34 (85)	33 (83)

n.a., not any nematode eggs detected on day 14 of albendazole treatment (FECR: 99.9%). Therefore, faecal cultures were not prepared

^a^Number of pools tested

### Genotypes of isotype 1 β-tubulin gene of *Haemonchus* spp.

**Fig. 2:**
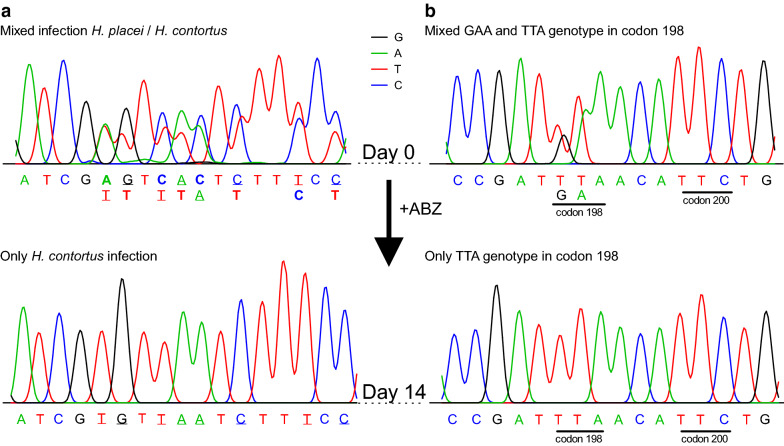
Typical effect of albendazole treatment on *Haemonchus* spp. species distribution and codon 198 polymorphisms. Pools of larvae from the same animals (5 animals less than one year old from Tulus) on day 0 (upper panels) and day 14 (lower panels) after treatment with albendazole (ABZ) were analysed for polymorphisms in the isotype 1 β-tubulin gene. The beginning of an intron (**a**) was used to determine the *Haemonchus* species composition. Bases specific for *H. placei* are printed in bold; bases specific for *H. contortus* are underlined. Another region further downstream in the chromatogram shows the sequences for codons 198 and 200 (**b**). Peaks specific for *H. placei* and for the susceptible genotype in codon 198 occurred only on day 0 but not on day 14

Sanger sequencing of PCR products was performed to identify the *Haemonchus* species harboured in each pool (L3 or adult) and to detect changes that might be involved in BZ resistance. All samples (*n *= 58), i.e. pooled L3 from trials (*n *= 40 pools of L3: 27 from day 0 and 13 from day 14 of albendazole treatment) in the five South Darfur study areas and pooled adult male *Haemonchus* spp. (*n *= 18) from abattoirs in Nyala, Rehed Al-Birdi and Tulus with unknown history of anthelmintic treatment, were genotyped (Table 5). Alignment of isotype 1 β-tubulin gene sequences from pooled adult male *Haemonchus* spp. identified the majority of the samples as *H. placei* (12/18) being, except for some sequencing errors at the very beginning or end, 100% identical to GenBank JQ342643. The remaining six samples contained a mixture of *H. contortus* and *H. placei* but the amount of *H. contortus* was apparently small since *H. contortus*-specific peaks were smaller than *H. placei*-specific peaks. Sequence alignment of pooled L3 samples on day 0 identified *H. placei* in 5 samples and *H. contortus* in 8 samples, and the remaining 14 samples were detected as mixed infections. All samples collected on day 14 (*n *= 13) were *H. contortus* only. The *H. contortus* samples were identical or almost identical to GenBank MN657178. In *H. placei* sequences, polymorphisms were not detected in any of the three codons. In samples identified as *H. contortus* or mixed infections, no polymorphisms were identified in codons 167 and 200 of the isotype 1 β-tubulin gene but exchanges were detected in codon 198. Wild-type amino acid glutamate (GAA) was identified in 67% of the samples (only day 0), leucine (E198L) in 28% samples, including all post-albendazole treatment samples, such as valine (E198V) in 4% and potentially stop codon TAA (E198Stop) in 2% samples. The E198V and E198Stop were detected only in day 0 samples. When comparing the sequence chromatograms of paired L3 samples before and after albendazole administration, mixed infections were detected before treatment whereas only *H. contortus* was identified after treatment (Fig. 2a). In parallel, the wild-type GAA (Glu) codon 198 was completely replaced by TTA (Leu) (Fig. 2b).

**Table 5:** Mutations in codon 198 in isotype 1 β-tubulin gene of *Haemonchus* spp. collected from cattle in five different study areas in South Darfur State, Sudan, detected by Sanger sequencing

Study area	Source of samples	Type of samples^a^	Sample collection day^b^	*Haemonchus* spp.	No.^c^	No. of samples with each variant			
						GAA	TTA	GTA	TAA
Beleil	Trial	L3	0	*H. placei*	0	0	0	0	0
				*H. contortus*	0	0	0	0	0
				Mixed infections^d^	1	1	0	0	0
			14	^e^	–	–	–	–	–
Kass	Trial	L3	0	*H. placei*	4	4	0	0	0
				*H. contortus*	0	0	0	0	0
				Mixed infections^d^	1	0	1	0	0
			14	*H. contortus*	1	0	1	0	0
Nyala	Trial	L3	0	*H. placei*	1	1	0	0	0
				*H. contortus*	0	0	0	0	0
				Mixed infections^d^	5	2	1	2	0
			14	*H. contortus*	2	0	2	0	0
	Abattoir	Adult	n.a.	*H. placei*	2	2	0	0	0
				Mixed infections^d^	1	1	0	0	0
Rehed Al-Birdi	Trial	L3	0	*H. placei*	0	0	0	0	0
				*H. contortus*	6	6	0	0	0
				Mixed infections^d^	4	4	0	0	0
			14	*H. contortus*	4	0	4	0	0
	Abattoir	Adult	n.a.	*H. placei*	3	3	0	0	0
				Mixed infections^d^	1	1	0	0	0
Tulus	Trial	L3	0	*H. placei*	0	0	0	0	0
				*H. contortus*	2	1	0	0	1
				Mixed infections^d^	3	2	1	0	0
			14	*H. contortus*	6	0	6	0	0
	Abattoir	Adult	n.a.	*H. placei*	7	7	0	0	0
				Mixed infections^d^	4	4	0	0	0
Total (and %)				*H. placei*	17	17 (100)	0 (0)	0 (0)	0 (0)
				*H. contortus*	21	7 (33)	13 (62)	0 (0)	1 (5)
				Mixed infections^d^	20	15 (75)	3 (15)	2 (10)	0 (0)
				All the samples	58	39 (67)	16 (28)	2 (4)	1 (2)

n.a., samples collected from cattle abomasa at abattoirs with unknown history of anthelmintic treatments

^a^Pools of third-stage larvae (L3) of at least ⁓ 1000 gastrointestinal nematode L3/sample or of adult male *Haemonchus* spp. isolated from cattle abomasa at abattoirs (16–100 worm/sample)

^b^Faecal samples were collected before treatment (day 0) and on day 14 after albendazole treatment at dose of 7.5 mg/kg body weight

^c^Number of pools tested

^d^Mixed infections with *Haemonchus placei*/*Haemonchus contortus* that identified in pools of L3 or adults based on chromatograms of the isotype 1 β-tubulin gene as described by Fávero et al. (submitted)

^e^No nematode eggs detected on day 14 of albendazole treatment (FECR: 99.9%). Therefore, faecal cultures were not prepared

Pyrosequencing assays were used to measure allele frequencies of resistance-associated genotypes at codon 167, 198 and 200 in isotype 1 β-tubulin gene of *H. placei* in pooled adult male samples (*n *= 8) from cattle abomasa at the abattoirs of Nyala, Rehed Al-Birdi and Tulus. Benzimidazole resistance allele-associated frequencies were 2.94%, 6.88% and 11.38% in codons F167Y, F200Y and E198A, respectively (Table 6).

**Table 6:** Mean resistance allele frequencies (%) ± standard deviation for the F167Y, E198A and F200Y single-nucleotide polymorphisms in isotype 1 β-tubulin gene of *Haemonchus placei* adult worms from cattle at three different study areas in South Darfur State, Sudan

Isolate	No. of pooled samples^a^	Codon 167 (TAC)	Codon 198 (GCA)	Codon 200 (TAC)^b^	Codon 200 (TAC)^c^
Nyala	2	3.00 ± 1.16	12.75 ± 3.10	6.75 ± 1.23	7.50 ± 1.73
Rehed Al-Birdi	3	2.67 ± 0.82	10.50 ± 0.84	5.50 ± 1.38	6.67 ± 0.82
Tulus	3	3.17 ± 0.75	11.33 ± 0.82	5.67 ± 0.52	6.67 ± 0.52
Total	8	2.94 ± 0.85	11.38 ± 1.78	5.88 ± 1.15	6.88 ± 1.03

For each sample, two technical replicates were analysed

^a^Pools of adult male *Haemonchus* spp. isolated from cattle abomasa at abattoirs (16–100 worm/sample) with unknown history of anthelmintic treatments

^b^Pyrosequencing assays conducted with the sequencing primer for codon 198

^c^Pyrosequencing assays performed with the codon 200 specific sequencing primer

## Discussion

Benzimidazoles are broadly administered anthelmintics used to treat infections with GINs in humans and animals [1, 6]. The heavy reliance on BZs (e.g. albendazole) in the control programmes, the small number of alternative anthelmintic compounds and the high rates of re-infections with GINs in endemic regions in different parts of the world have resulted in the emergence of BZ resistance among GIN populations that infect animals [6, 38]. Furthermore, there is also suspicion that parasitic nematodes of humans are also at risk of evolving such resistance [39]. Benzimidazole resistance is a significant problem in veterinary medicine, particularly in GINs of sheep and goats. Resistance to BZs has been intensively studied around the world in small ruminants, but in cattle relatively few studies have been published [9, 40, 41]. *Haemonchus contortus* has been demonstrated around the world as the predominant BZ-resistant nematode, while few studies have reported resistance of *H. placei* [11, 42]. The molecular mechanisms of BZ resistance in strongylid nematodes, including *H. contortus* and *H. placei*, have been correlated with the occurrence of mutations in three different codons in the isotype 1 β-tubulin gene leading to the exchanges F167Y, E198A and F200Y [12–14, 43]. Recently, additional mutations were reported for codon 198 of isotype 1 β-tubulin gene of *H. contortus*, *T. circumcincta* and *Trichostrongylus axei* including E198L, E198V, E198K, E198I and E198Stop [15–17, 19].

There have been a few published reports on BZ resistance at the phenotypic level in cattle in Africa, including Kenya and Mali [44, 45]. The present study was designed to obtain insight into the efficacy of albendazole and the resistance status to BZs in cattle parasitic nematodes in South Darfur based on phenotypic and molecular techniques. Herein, molecular biology was employed to detect the presence of the economically most important nematodes pre- and post-albendazole treatment, identify the occurrence of single or mixed infections with *H. contortus* and *H. placei*, and the presence of exchanges at codons 167, 198 and 200 in the isotype 1 β-tubulin gene of *H. contortus* or *H. placei*. Reduced efficacy of albendazole was observed in three out of the five South Darfur study areas. In Nyala, Rehed Al-Birdi and Tulus the FECRs were 94.6%, 92.0% and 88.5% on day 14. The EC_50_ in the EHT supported the above findings. In the three study areas, the EC_50_ was higher (0.032–0.037 µg/ml thiabendazole) than in Beleil and Kass (0.011–0.012 µg/ml thiabendazole) where albendazole was found to be fully effective. In Tulus, the EC_50_ using nematode eggs of day 14 of albendazole treatment was slightly higher (0.113 µg/ml thiabendazole) than the cut-off value of BZ resistance (0.1 µg/ml thiabendazole) [23]. The paired data of day 8 and 14 clearly indicated the development of BZ resistance in cattle in Tulus [FECRT: 90.9% (95% CIs: 89.6–92.3%) and 88.5% (95% CIs: 87.2–90.1%) for day 8 and 14, respectively], since the FECRs and the respective upper and lower 95% CIs were below the set criteria for BZ resistance by WAAVP [29]. The paired results of Rehed Al-Birdi were inconclusive, and a slightly reduced efficacy (not significant) to BZs was shown in Nyala. This is the first report of BZ resistance in cattle in Sudan. This finding is supported by few previous reports from parts of Africa. In Kenya, a field trial in smallholder dairy cattle showed 74.9% efficacy to albendazole [44]. Furthermore, a study from southeast Mali reported 79.3% efficacy to albendazole in cattle naturally infected with GINs [45].

The data of the FECRT of this study were analysed on day 8 and 14 post-treatment and paired analyses were compared to unpaired comparison of a treatment with a control group. The results revealed that the paired data analysis using sampling on day 14 was more powerful in detection of BZ resistance development in cattle in Sudan than paired analysis with samples on day 8 post-treatment or unpaired analysis. This suggestion is supported by the recommendation of the upcoming new (unpublished) WAAVP guideline as presented at the WAAVP conference 2019 [46]. Both phenotypic tests, FECRT and EHT, were found to be highly correlated (*ρ* = − 1; *p* = 0.017) in detection of BZ resistance in cattle in South Darfur. Since FECRT is labour and thus cost intensive, we employed the EHT as an alternative sensitive technique [47] to examine BZ efficacy in cattle in Sudan. In the present study, areas identified as exhibiting GIN populations susceptible to BZs showed a FECR of 99.5–99.9% (paired results of day 14) and an EC_50_ of 0.011–0.012 µg/ml thiabendazole, while in areas with populations showing reduced or suspected reduced albendazole efficacy the FECRT was in a range of 88.5–94.6% and the EC_50_ ranged between 0.032 and 0.037 µg/ml thiabendazole, which would all correspond to susceptible populations applying a threshold for BZ resistance of 0.1 µg/ml thiabendazole [23]. When these phenotypic findings (FECRT and EHT) are compared to previously published field work on mixed species samples, the EC_50_ values of BZ-susceptible nematodes in South Darfur were below the EC_50_ values of BZ-susceptible nematodes of cattle in Germany (0.027–0.046 µg/ml thiabendazole) [47, 48]. Furthermore, the EC_50_ values of GIN populations in South Darfur where they showed potentially reduced albendazole efficacy were in the range of the previously mentioned BZ susceptible values from Germany. Since the FECRT and the EHT were found to be highly correlated (Fig. 1), the EHT data apparently do represent the resistance status adequately despite the fact that only populations in one region demonstrated BZ resistance and in two others reduced efficacy was observed and the level of resistance was apparently low. Therefore, the differences in the EC_50_ values between GIN populations in Sudan and in other regions might be related to the behaviour of the parasites, the intensity of nematode infections and the different climates. Further investigations using eggs extracted from different cattle parasitic nematodes derived from different geographical regions will be required to help to establish whether the EC_50_ cut-off value for BZ resistance in cattle GIN needs to be adjusted for certain geographical regions.

To allow highly sensitive qualitative diagnosis of the economically most important GINs in cattle pre- and post-treatment with albendazole, genus-specific PCRs were used. Test of pooled L3 samples on day 0 did not detect the presence of *Teladorsagia* spp. and *Ostertagia* spp., while *Haemonchus* spp., *Trichostrongylus* spp. and *Cooperia* spp. were identified. When L3 samples on day 14 post-albendazole treatment were examined, *Haemonchus* spp. was identified in all samples (*n *= 13), while *Trichostrongylus* spp. and *Cooperia* spp. were observed in 62% and 54% of the samples, respectively. These findings are in agreement with our previous study in goats in South Darfur using the same technique [19] showing that the majority of egg-shedding post-albendazole treatment was due to *H. contortus*. *Trichostrongylus* spp. and *Cooperia* spp. survived treatment suggesting the development of BZ resistance in these parasites, but at a low level. In Sudan, no previous data for anthelmintic efficacy in cattle are available, and few studies were published from other African countries, which mostly reported no development of anthelmintic resistance in cattle [49, 50]. Albendazole efficacy was reported as 95.5% FECR in some cattle farms in Ethiopia [50], 74.9% and 98% in two different studies from cattle in Kenya [44, 49]. All mentioned three studies, as well as most published reports from Africa, were based on the use of the McMaster method as a quantitative tool for nematode egg counts and the microscopic differentiation of nematode L3 pre- and post-anthelmintic treatment. The sensitivity of the McMaster technique is comparatively low and only able to detect anthelmintic resistance when at least 25% of the nematode population is resistant [26, 51]. Herein, the use of the Mini-FLOTAC technique enhanced the sensitivity of the FECRT. Moreover, the highly sensitive genus-specific PCRs allowed detection of *Haemonchus* spp., *Trichostrongylus* spp. and *Cooperia* spp. after albendazole treatment although microscopic differentiation of larvae detected only *Haemonchus* spp.

To identify the molecular mechanisms of BZ resistance in cattle nematodes in South Darfur, Sanger sequencing and pyrosequencing of isotype 1 β-tubulin genes of *H. contortus* and *H. placei* were performed using DNA (*n *= 58) from pooled L3 samples during trials and from pooled adult male *Haemonchus* spp. samples isolated from cattle abomasa collected at abattoirs. Sanger sequencing of all pooled adult male *Haemonchus* spp. (*n *= 18) revealed that samples were *H. placei* and mixed infections containing small amounts of *H. contortus*. The sequence chromatograms of these samples did not detect any polymorphisms at codon positions 167, 198 and 200. Sequences of L3 samples (*n *= 40: 27 from day 0 and 13 from day 14 of albendazole treatment) identified only *H. placei* in five samples from day 0 in Kass and Nyala with no detection of any polymorphisms in the three codons. Furthermore, comparison of sequence chromatograms of paired L3 samples before and after treatment with albendazole indicated that *H. placei* was susceptible to BZs in South Darfur State since post-treatment only *H. contortus* was detectable (Fig. 2a). Pyrosequencing assays confirmed the above results and detected no higher BZ resistance-associated allele frequencies (≤ 11.38%) than the technical background [10% [35]] in *H. placei*. This finding is in agreement with a previous study from Nigeria describing that pyrosequencing assays conducted on *H. placei* isolated from cattle at abattoirs of six different states did not show any evidence of BZ resistance-associated alleles and identified only susceptible genotypes [35]. Sanger sequencing of the remaining 22 pooled L3 samples of day 0 identified exclusively *H. contortus* in 8 samples and 14 samples as mixed *H. contortus/H. placei* infections. The day 14 L3 samples (*n *= 13) contained only *H. contortus* but no *H. placei*. While no polymorphisms were detected in codon positions F167Y and F200Y, mutations were identified in codon 198 of isotype 1 β-tubulin gene of *H. contortus*, i.e. E198L [16/all sequenced samples, L3 and adults (*n *= 58)], E198V (2/58) and potentially E198Stop (1/58). The substitution E198L was identified in 100% of L3 samples on day 14 suggesting a strong correlation between the occurrence of this mutation and the development of BZ resistance in Sudan (Fig. 2b). This is in agreement with our recent results for *H. contortus* collected from goats [19] demonstrating the presence of E198L in all pooled L3 and adults surviving albendazole treatment and a large increase of peak intensities for bases coding for leucine when the sequence chromatograms of day 0 were compared to day 14 after albendazole treatment. The same exchange has been previously described in a highly multi-resistant *T. circumcincta* isolate [52]. In a very recent study from Spain, the frequency of E198L (GAA/CTA) was found to be highly associated with the response to BZ treatment in sheep naturally infected with *T. circumcincta*. In this study, the frequency of leucine was changed from the range of 10.4–80.7% in samples before treatment to a range of 82.3–92.8% after treatment [53]. In a very important new publication, leucine, alanine and valine were introduced at codon position 198 of the *ben-1* β-tubulin isotype into the BZ susceptible, free-living Bristol N2 *Caenorhabditis elegans* using CRISPR/Cas-9 mediated genome editing followed by exposure to different concentrations of albendazole and fenbendazole. This study proved for the first time, to our knowledge, that all three exchanges conferred equal levels of BZ resistance (also equal to F167Y and F200Y) but also showed that E198V was associated with decreased fitness at least in *C. elegans* under the given laboratory conditions [18]. The decreased fitness might explain the low frequency of E198V in field populations of *T. circumcincta* and *H. contortus*. However, there is no formal proof of this causality and to exclude whether there are other effects contributing to the low frequency of E198V. It must be kept in mind that there are in general considerable differences between the β-tubulin repertoires of *C. elegans* and strongyles such as *H. contortus* [54, 55]. In *C. elegans*, loss of function mutations in the β-tubulin paralogs *Cel-ben-1* are sufficient to confer resistance to BZs [56]. The β-tubulin paralogs *Cel-tbb-4*, *Cel-tbb-6* and *Cel-mec-7* encode phenylalanine at the position of codon 167 and 200 as well as glutamate in position 198 and they are therefore BZ susceptible [57]. However, the tubulin paralogs *Cel-tbb-4* and *Cel-mec-7* are not widely expressed and have more specialized functions in a subset of sensory neurons [58, 59], while not much is known about tbb-6 except that RNA interferences causes only a minor negative effect on the life span of worms [60]. The other two β-tubulin genes with high expression levels in many tissues encode a tyrosine in codon 200 and are therefore expected to be resistant to the effects of BZs. In contrast, there are at least two highly expressed β-tubulin paralogs in strongyles that are expected to be susceptible to BZs [13, 61]: In *H. contortus*, the isotype 1 β-tubulin gene has been commonly associated with BZ resistance, but also the isotype 2 β-tubulin gene has been implicated in resistance to BZs a few times [54, 61]. Both genes (isotype 1 and 2) have a paralogous relationship and are more closely related to *Cel-ben-1* than other *C. elegans* β-tubulins [10]. Despite these differences, the results of the recent genome editing study in *C. elegans* [18] provide novel fundamental insights into the mechanisms of BZ resistance in clade V nematodes.

In general, E198L was found to be widespread in *T. circumcincta* and *T. axei* in sheep farms in the UK [17] but in Ireland only in *T. circumcincta* from one sheep farm [62]. In the UK, E198L allele frequencies were 91.7% in one farm, but high frequencies of F200Y were observed on other farms [15, 17]. In Brazil, the E198L substitution was found in *H. contortus* in one out of eight samples from sheep [16]. To the knowledge of the authors, the present study is the first to identify the E198L substitution in nematodes collected from cattle and is the second report of an E198L substitution in *H. contortus* that were in parallel shown to be phenotypically resistant to BZs. Detection of E198V for the second time in South Darfur together with the similar physicochemical properties of alanine, leucine and valine and in particular the results obtained by genome editing of the *C. elegans ben-1* gene [18] strongly suggest that this mutation is associated with BZ resistance in field populations from Sudan. Although a loss-of-function allele of the β-tubulin gene *ben-1* has been shown to confer BZ resistance in the model nematode *C. elegans* [63], it is quite unlikely that such an exchange as E198Stop can have a similar effect in *H. contortus*. In the presence of a drug-susceptible isotype 2 paralog, a loss of function allele should not result in resistance. However, since the genotype of the isotype 2 in the same individuals is not known, further research including genotyping the isotype 2, as previously reported for *Trichostrongylus colubriformis* [64], and confirmation or exclusion of the presence of a stop codon, e.g. by deep amplicon sequencing, is needed.

Accurate differentiation of nematode species is of interest not only for diagnosis, treatment and control but also for epidemiological studies. It is well known that *H. contortus* and *H. placei* are separate species based on morphological and molecular characters. *Haemonchus contortus* frequently infects sheep and goats, while *H. placei* predominantly infects cattle [4, 40]. Mixed infections with small ruminant and cattle nematodes in one host has been described previously in some regions, e.g. Brazil and Pakistan [42, 65, 66]. In the present study, *H. contortus* was detected in pooled L3 samples and at low level in some pooled adult male *Haemonchus* spp. samples from cattle at the abattoirs. It is likely that *H. contortus* in cattle were derived from pastures contaminated by infected sheep and goats. In South Darfur, farmers graze calves with sheep and goats, while cattle graze in a separate group for milk production (Mohammedsalih, personal observation). As BZ-resistant *H. contortus* were found to be common in goats in Nyala, Rehed Al-Birdi and Tulus [19, 20], it is probable that this was the source of the resistant worms in the calves. Given the large numbers of *H. placei* and the low numbers of *H. contortus* in the calves, it would be very likely that detection of BZ resistance in such populations fails. The farming practices in South Darfur are critical not only for spreading BZ-resistant GIN populations between cattle and small ruminants, but also might be raising the possibility of interspecies hybridisation in *H. contortus* and *H. placei* in field populations. This suggestion is supported by previous studies which identified hybrid *H. contortus* × *H. placei* after experimental co-transplantation of adult worms into the abomasum of a recipient sheep [67, 68] and also detected hybrids of both species in field isolates from cattle in Pakistan [42, 66]. Presence of hybrid species in the field could lead to introgression of resistance alleles from *H. contortus* into *H. placei* if these hybrids are fertile. This would provide a mechanism for passing anthelmintic resistance from *H. contortus* to *H. placei* [40]. Introgression of genes between BZ-resistant *H. contortus* (E198L) and susceptible *H. placei* in fields of South Darfur would be a major concern and of general interest for the prevalence of anthelmintic resistance in the two different *Haemonchus* species.

Screening the animals for presence of infections with gastro-intestinal helminths can provide some epidemiological information. Since the five study areas were visited only in one season (autumn) and parameters such as local temperatures, humidity and weight of the animals were not recorded, the results of risk factor analysis are not representative for all cattle in South Darfur. Nevertheless, the results provide some preliminary insights into geographical information and correlation of the infection rate with animal age and sex. The results of the present study indicate that infection with gastro-intestinal helminths is commonly occurring in cattle in South Darfur (71%). These findings are in agreement with our recent study in goats in South Darfur [20] and with the few previously published studies in cattle from Sudan [69, 70]. Risk factor analysis showed different infection levels among the five study areas. Animals in Rehed Al-Birdi and Tulus shed significantly higher numbers of strongyle eggs than those in Kass. This may have been related to the level of precipitation and the time of sample collection, i.e. in the beginning, middle or late part of the rainy season. From the present study, age of the animal and the sex were identified as important factors associated with gastro-intestinal strongyle infections in cattle in South Darfur. These findings are consistent with findings from previous studies in Kenya and Zimbabwe [71, 72].

## Conclusions

Benzimidazole-resistance in GINs is a putatively emerging threat for the health of cattle in Sudan. *Haemonchus* spp. were found to be the predominant nematodes surviving albendazole treatment while BZ-resistant *Cooperia* spp. and *Trichostrongylus* spp. were also detected. *Haemonchus placei* was susceptible to BZs, while BZ resistance in cattle GIN was due to mixed infections with *H. contortus*. Polymorphisms were detected at codon 198 in the isotype 1 β-tubulin gene of *H. contortus*, including E198L, E198V and potentially E198Stop. The E198L substitution was identified in 100% of the samples that survived albendazole treatment. Additional studies on the effects of the three codon 198 substitutions in model systems will be required to understand the exact molecular mechanisms of BZ resistance in Sudan. Further large-scale epidemiological studies in South Darfur will be required to monitor the level of mixed infections in cattle with the two species of *Haemonchus*.

## Supplementary Information

**Additional file 1: Table S1**. Arithmetic means of egg counts (and 95% confidence interval) with cattle naturally infected with strongyle nematodes before and after oral administration of albendazole at dose of 7.5 mg/kg body weight to the treated groups at five different study areas in South Darfur State, Sudan

## Data Availability

The relevant information has been included in the manuscript. Data analysed for this manuscript are available from the corresponding author on request.

## Abbreviations

BZs: Benzimidazoles
CIs: Confidence intervals
EC_50_: Concentration of thiabendazole that inhibited 50% of larvae hatching
EHT: Egg hatch test
epg: Eggs per gram
FECRT: Faecal egg count reduction test
GINs: Gastro-intestinal nematodes
L3: Third-stage larvae
WAAVP: World Association for the Advancement of Veterinary Parasitology
