# Supplementary material for: Susceptible trichostrongyloid species mask presence of benzimidazole-resistant Haemonchus contortus in cattle
Source: Parasit Vectors. 2021 Feb 8;14:101. doi: 10.1186/s13071-021-04593-w (PMC7869217; doi:10.1186/s13071-021-04593-w)
Supplement: Supplementary file 1 — Additional file 1: Table S1. Arithmetic means of egg counts (and 95% confidence interval) with cattle naturally infected with strongyle nematodes before and after oral administration of albendazole at dose of 7.5 mg/kg body weight to the treated groups at five different study areas in South Darfur State, Sudan [file 13071_2021_4593_MOESM1_ESM.docx]

**Table S1**

Arithmetic means of egg counts (and 95% confidence interval) with cattle naturally infected with strongyle nematodes before and after oral administration of albendazole at dose of 7.5 mg/kg body weight to the treated groups at five different study areas in South Darfur State, Sudan.

| **Study area** | **Groups** | **Mean (95% CI)** |  |  |
| --- | --- | --- | --- | --- |
|  |  | **Day 0** | **Day 8** | **Day 14** |
| Beleil | Control: *n*=6 | 283 (143 – 424) | 198 (92 – 305) | 187 (89 – 284) |
|  | Treated: *n*=11 | 327 (241 – 414) | 0^a,b^ | 0^a,b^ |
| Kass | Control: *n*=5 | 303 (217 – 389) | 338 (254 – 423) | 391 (319 – 463) |
|  | Treated: *n*=11 | 309 (208 – 409) | 1^a,b^ (-1 – 3) | 1^a,b^ (-2 – 4) |
| Nyala | Control: *n*=10 | 329 (251 – 407) | 251 (168 – 335) | 257 (173 – 342) |
|  | Treated: *n*=20 | 355 (290 – 419) | 6^a,b^ (1 – 11) | 9^a,b^ (1 – 18) |
| Rehed Al-Birdi | Control: *n*=10 | 1014 (369 – 1659) | 982 (33 – 1931) | 744 (188 – 1300) |
|  | Treated: *n*=20 | 1245 (653 – 1837) | 47^a,b^ (-28 – 122) | 56^a,b^ (-30 – 142) |
| Tulus | Control: *n*=10 | 513 (67 – 958) | 707 (-55 – 1469) | 732 (-128 – 1592) |
|  | Treated: *n*=20 | 790 (342 – 1237) | 48^a,b^ (-10 – 105) | 68^a,b^ (-24 – 161) |

^a^ Significantly different (p<0.05) to control on the same day using a Kruskal-Wallis test with Dunn’s post hoc test

^b^ Significantly different (p<0.05) to day 0 in the same group using a Friedman test with Dunn’s post hoc test

⸆ Significantly different (p<0.05) to day 8 in the same group using a Friedman test with Dunn’s post hoc test
